# Serum prolidase level in patients with brucellosis and its possible relationship with pathogenesis of the disease: a prospective observational study

**DOI:** 10.3906/sag-1902-122

**Published:** 2019-10-24

**Authors:** Oğuzhan Sıtkı DİZDAR, Ayşe TURUNÇ ÖZDEMİR, Osman BAŞPINAR, Derya KOÇER, Yavuz KATIRCILAR, İlhami ÇELİK

**Affiliations:** 1 Department of Internal Medicine and Clinical Nutrition, Kayseri City Training and Research Hospital, Kayseri Turkey; 2 Department of Clinic Microbiology and Infectious Disease, Kayseri City Training and Research Hospital, Kayseri Turkey; 3 Department of Internal Medicine, Kayseri City Training and Research Hospital, Kayseri Turkey; 4 Department of Biochemistry, Kayseri City Training and Research Hospital, Kayseri Turkey

**Keywords:** Prolidase, brucellosis, pathogenesis, collagen, treatment

## Abstract

**Background/aim:**

Changes in collagen metabolism and fibroblastic activity may play a role in the pathogenesis of brucellosis. The prolidase enzyme plays an important role in collagen synthesis. We aimed to investigate the association of prolidase levels with brucellosis.

**Materials and methods:**

Serum prolidase levels in 20 patients newly diagnosed with brucellosis were compared with levels in 30 healthy control subjects. Patients with brucellosis were reassessed 3 months later for prolidase, other laboratory measurements, and response to treatment.

**Results:**

The levels of serum prolidase were significantly higher in brucellosis patients compared with those of healthy controls. Prolidase, sedimentation, and C-reactive protein levels were significantly lower after antibrucellosis treatment than before treatment.

**Conclusion:**

The current study is the first to demonstrate significantly increased serum prolidase levels in patients with brucellosis compared with healthy controls. Prolidase levels also significantly decreased with antibrucellosis treatment. This finding provides a new experimental basis to understand the pathogenesis of brucellosis in relation to collagen metabolism. The increase in serum prolidase levels might be related to several factors such as tissue destruction, increased fibroblastic activity, and granuloma formation, all of which are involved in the natural history of brucellosis.

## 1. Introduction

The clinical presentation of brucellosis is nonspecific and the course of infection is variable. Brucellosis presents as a multisystem disease involving many organs and tissues [1]. The mechanisms underlying the manifestations of brucellosis are not completely understood. Furthermore, biopsied samples of tissues in patients with brucellosis may show noncaseating granulomas, but the molecular mechanism underlying this change remains unclear. 

Prolidase is a cytosolic exopeptidase that splits imidodipeptides with C-terminal proline or hydroxyproline. This enzyme has a major role in recycling proline from imidodipeptides for collagen resynthesis and cell growth. Therefore, prolidase is considered to be a limiting factor in the regulation of collagen production [2]. Prolidase activity has been reported in leukocytes, erythrocytes, plasma, and various organs such as the brain, heart, kidney, uterus, thymus, and dermal fibroblasts [3]. Serum prolidase enzyme activity is elevated in conditions that are characterized by chronic inflammation of tissue and/or increased turnover of collagen. We hypothesized that serum prolidase levels may be associated with brucella infection, brucellosis-related tissue damage, and granuloma formation.

In this study, we explored prolidase levels in patients with brucellosis and healthy controls. We revealed the relationship between the prolidase level and changes in clinical status and disease activity in order to clarify the role of prolidase in the pathogenesis of brucellosis. To our knowledge, there is no published data regarding prolidase levels in patients with brucellosis. 

## 2. Materials and methods

### 2.1. Study design and participants

This prospective study included 20 patients who were newly diagnosed with brucellosis recruited from the internal medicine and infectious disease clinic of our hospital from January 2017 to December 2017. Thirty sex-matched healthy controls (HC) who visited our hospital in the same period were enrolled in this study. HCs did not have any history of brucellosis. Patients with brucellosis were reassessed 3 months later for prolidase measurement and response to treatment. We did not have data loss during the follow-up of the patients with brucellosis. Written informed consent was provided by each participant, and the study protocol was approved by the Local Ethics Committee.

The exclusion criteria for patients with brucellosis were as follows: history of malignant cancers, concomitant presence of any inflammatory disease, any rheumatic diseases, any endocrine disease (diabetes mellitus, parathyroid or thyroid disease), or chronic renal or hepatic disease, as well as patients who were currently receiving antibrucellosis therapy.

Healthy controls were volunteers who had no evidence of acute or chronic infectious disorders, autoimmune disease, or any other systemic condition.

Clinical data from each patient including age, sex, occupation, residence, transmission route, symptoms at diagnosis, physical examination findings, weight, height, and current medications were recorded. Laboratory assessment included complete blood count, C-reactive protein (CRP), sedimentation (ESR), and liver and kidney function tests. Blood specimens were taken from each patient following an overnight fast before the antibrucellosis therapy and 3 months after treatment. Serum was stored at −80 °C until analysis for prolidase level.

### 2.2. Diagnosis of brucella

Cases who had musculoskeletal pain, fever of unknown origin, and acute or chronic arthritis were suspected to have brucellosis according to their clinical manifestations. The diagnosis of brucellosis was established for patients presenting with symptoms suggestive of brucellosis according to the presence of one of the following criteria;

1. Wright titer of equal or greater than 1/160 and 2-mercaptoethanol (2ME) test of ≥1/80.

2. Brucellosis-positive blood, bone marrow, and synovial fluids culture.

Relevant demographic, clinical, and laboratory data and treatment modalities and outcomes were obtained from patients’ follow-up cards and hospital records.

### 2.3. Measurement of serum prolidase level

Serum prolidase level was measured by double-antibody sandwich technique using an enzyme-linked immunosorbent assay (ELISA) kit (Shanghai Sunred Biological Technology Co. Ltd., Shanghai, China). Assay range was 0.5–150 ng/mL, and intraassay CV was <10%. The concentrations of the samples were calculated through calibration curves obtained from study standards with known levels. Serum prolidase level was expressed as ng/mL.

### 2.4. Statistical analysis

Data are expressed as mean ± standard deviation (SD) or number (percentages). The normality and the homogeneity of the data were examined by the Shapiro–Wilk test and Levene test, respectively. Comparisons between groups for continuous variables were performed using Student’s t-test (normal distribution) or the Mann–Whitney U test (nonnormal distribution). The Fisher test or the χ2 test was used for all categorical data. A paired sample t-test was conducted to compare the two measurements of patients for prolidase levels at baseline and 3 months later. For all calculations, SPSS statistical package (version 15.0; SPSS, Chicago, IL, USA) was used. P < 0.05 was considered statistically significant.

## 3. Results

We included 20 patients newly diagnosed with brucellosis and 30 healthy controls in this study. In the case group, 8 were male (40%) and 12 were female (60%), with a mean age of 48.5 ± 15.4 years. Doxycycline and streptomycin were the most frequently used therapeutic regimens (95%) in the patients. One patient received trimethoprim sulfamethoxazole and doxycycline because of ototoxicity and liver function test abnormalities due to previous treatment. All patients received treatment for at least 6 weeks. Only one patient received treatment for 12 weeks. Relapse occurred in only one patient after treatment. Hematological findings were pancytopenia (10%) and thrombocytopenia (5%). Osteoarticulary findings of the patients were as follows: 1 patient had spondylodiscitis, 2 patients had sacroiliitis, and 1 patient had arthritis. 

The demographic data of the patients with brucellosis and the control group are shown in Table 1. There were no significant differences in sex between groups; however, the ages of patients were significantly higher than those of the controls (P = 0.001). There were significant differences between patients with brucellosis and healthy controls with respect to prolidase level. The serum prolidase level was significantly higher in patients with brucellosis than in the control group (P < 0.001). The patients were classified as having acute, subacute, or chronic brucellosis according to the duration of their disease: <8 weeks, 8–52 weeks, and >52 weeks, respectively. There were 9 patients with acute, 6 with subacute, and 5 with chronic brucellosis. There was no statistically significant difference in serum prolidase levels among acute, subacute, and chronic cases with brucellosis (85.9 ng/mL, 110.2 ng/mL, and 114.8 ng/mL, respectively; P = 0.332). The clinical characteristics of the patients with brucellosis are summarized in Table 2. 

**Table 1:** The demographic data and prolidase level of the patients with brucellosis and control group.

Variable	Control group(n = 30)	Patients with brucellosis(n = 20)	P-value
Age	34.1 ± 8.1	48.5 ± 15.4	0.001
Sex, F/M	12 (40)/18 (60)	8 (40)/12 (60)	1.000
Prolidase level, ng/mL	40.8 ± 8.9	100.4 ± 38.9	<0.001

F: female, M: male.

**Table 2:** Clinical characteristics of patients with brucellosis.

Variables	Patients with brucellosis(n = 20)
Occupation, n (%)	
livestock ranger	10 (50)
house wife	4 (20)
veterinarian	1 (5)
other	5 (25)
Residence	
rural area	10 (50)
city	10 (50)
Transmission route	
nonpasteurized milk products	11 (55)
animal contact	4 (20)
nonpasteurized milk products and animal contact	5 (25)
Symptoms at diagnosis	
fatigue	20 (100)
sweating	20 (100)
arthralgia	18 (90)
fever	13 (65)
lumbago	11 (55)
weight loss	3 (15)
lack of appetite	17 (85)
nausea	3 (15)
headache	5 (25)
Hepatomegaly	4 (20)
Splenomegaly	2 (10)
Osteoarticular findings	5 (25)

Table 3 summarizes the serum prolidase levels, ESR, CRP, and other laboratory parameters of patients with brucellosis before and 3 months after antibrucellosis treatment. Prolidase levels, ESR, and CRP were significantly lower after antibrucellosis treatment than before treatment. Only 1 patient relapsed after treatment, and the prolidase level of that patient was higher than the pretreatment level (135.6 ng/mL and 151.5 ng/mL, respectively).

**Table 3:** Serum prolidase levels and other laboratory parameters of patients with brucellosis before and after 3 months of antibrucellosis treatment.

Variables	Before treatment	After treatment	P-value
Prolidase, ng/mL	100.4 ± 38.9	88.1 ± 46.4	0.049
C-reactive protein, mg/L	22.6 ± 43.5	3.7 ± 1.5	0.001
White blood cell count, 103/µL	8.93 ± 4.42	7.18 ± 1.67	0.204
Hemoglobin, g/dL	14.6 ± 1.6	14.9 ± 1.7	0.398
Sedimentation, mm/hour	12.7 ± 11.3	7.4 ± 7.1	0.002
AST, U/I	52.1 ± 75.9	37.8 ± 59.1	0.351
ALT, U/I	50.8 ± 56.1	45.2 ± 85.6	0.058
Platelets, 103/µL	252 ± 91	247 ± 70	0.823
Blood urea nitrogen, mg/dL	15.1 ± 4.3	14.9 ± 8.3	0.176

AST: aspartate aminotransferase, ALT: alanine aminotransferase.

## 4. Discussion

The prolidase enzyme, which has a role in the final step of collagen breakdown, is present in various tissues and plasma [3]. This enzyme plays an important role in the recycling of proline for collagen synthesis and cell growth [4]. During protein catabolism, prolidase catalyzes the degradation of intracellular collagen; its activity may be correlated with the rate of collagen degradation [5]. Due to the fact that brucella is a disease that affects many systems, it is very likely that it affects the metabolism of collagen in many tissues of the body [6]. Based on these data, the presence of a significant association between prolidase level and brucellosis can be considered.

Changes in prolidase activity may play important roles in extracellular matrix turnover, and therefore may also play a role in the development and outcome of several diseases [7,8]. Prolidase enzyme activity has been investigated in various disorders [9–12]. An increase or decrease in prolidase activity can demonstrate the existence of a disease state as well as the progression of the condition. Although some authors suggest that prolidase activity decreases in some disease conditions, such as asthma [13], chronic obstructive pulmonary disease [14], and ankylosing spondylitis [15], increased prolidase activity has been reported in some other diseases and cancers (pancreatic cancer, lung carcinoma, breast cancer) [16–19]. In subjects with chronic liver disease (chronic hepatitis B and C) and nonalcoholic steatohepatitis, serum prolidase activity has been shown to increase, especially in the early stage of fibrosis [20–23]. To the best of our knowledge, there is no existing data concerning the serum prolidase levels in patients with brucellosis.

The current study is the first to demonstrate significantly increased serum prolidase levels in patients with brucellosis compared with healthy controls. This finding suggests that brucellosis causes collagen tissue damage and increased collagen turnover; therefore, prolidase enzyme activity is increased in brucellosis. The present study is also the first to evaluate both prolidase levels and disease activity. We found that the prolidase levels significantly decreased with antibrucellosis treatment. These results demonstrated that the pathogenesis of brucellosis could be elucidated by the collagen metabolism. Synovial fibroblast may play an important role in the pathogenesis of osteoarticular diseases in brucellosis [24]. Fibroblastic activity during the natural history of brucellosis might be another contributing factor for increased serum prolidase level. 

Brucellosis has both acute and chronic properties of inflammation. Therefore, increased CRP and ESR have been reported to be involved in active disease and are often considered as useful criteria for the diagnosis and follow-up on the effectiveness of treatment in brucellosis [1,25]. In our study, the highest levels of prolidase, ESR, and CRP were seen in the patients with acute brucellosis. However, prolidase, ESR, and CRP levels decreased significantly with treatment. This result suggests that prolidase may be an important parameter in evaluating the response to treatment and may be used as a possible indirect inflammatory marker in patients with brucellosis. 

Host protease disorders can cause granuloma formation, leading to collagen degradation and tissue damage. Myara et al. demonstrated higher plasma prolidase activity in the early stage of chronic liver disease, indicating its role in extracellular matrix formation [9]. In different studies, higher serum prolidase activities were found in patients with pulmonary tuberculosis, especially in patients with lung cavities [26,27]. It has been suggested that increased prolidase levels might be related to tissue damage and enhanced fibroblast activity. In patients with brucellosis, biopsy of the affected organ may reveal noncaseating granulomas. Therefore, we can speculate that increased serum prolidase levels might be related to tissue damage, enhanced fibroblastic activity, and granuloma formation. Future studies which include tissue biopsy may give better understanding of this situation.

Our study has several limitations. First, it had a relatively small sample size. Second, we did not perform tissue biopsy. It would be very useful to be able to detect the formation of granuloma and other pathological findings. However, this is a preliminary study providing information regarding collagen metabolism in patients with brucellosis by evaluating serum prolidase levels. 

We revealed increased prolidase levels in patients with brucellosis. This finding provides a new experimental basis for understanding the pathogenesis of brucellosis. The increase in serum prolidase levels might be related to several factors such as tissue destruction, increased fibroblastic activity, and granuloma formation, all of which are involved in the natural history of brucellosis. This may be interpreted as evidence of increased collagen turnover in brucellosis. Monitoring serum prolidase level in clinical practice may be a suitable and useful method for evaluating patients with brucellosis. Further prospective studies are required to cross-validate our findings in larger cohorts of patients with brucellosis.
